# Application of a targeted and quantificational foraminoplasty device in percutaneous transforaminal endoscopic discectomy for L5–S1 disc herniation: preliminary clinical outcomes

**DOI:** 10.1186/s13018-021-02533-z

**Published:** 2021-06-22

**Authors:** Jinlong Liu, Junlong Wu, Honglei Zhang, Rui Zuo, Jiabin Liu, Chao Zhang

**Affiliations:** 1 Department of Spine Surgery, People’s Hospital of Liaocheng, Liaocheng, 252000 Shandong China; 2 Department of Orthopaedics, The 941th Hospital of Joint Logistics Support Force of Chinese PLA, Xining, 810000 Qinghai China; 3 Department of Orthopaedics, The Hospital of People Liberation Army Hong Kong Garrison, Hong Kong, 999077 China; 4 Department of Spine Surgery, Xinqiao Hospital, Army Medical University, Chongqing, 400037 China

**Keywords:** Percutaneous transforaminal endoscopic discectomy, Lumbar disc herniation at the L5–S1 level, Foraminoplasty, Preliminary clinical outcomes

## Abstract

**Objective:**

Percutaneous transforaminal endoscopic discectomy (PTED) is minimally invasive and has been widely used to treat patients with lumbar disc herniation (LDH) due to its safety and efficiency. However, due to the unique anatomy of the L5–S1 level, the PTED procedure is often difficult to perform in the region. ZESSYS, a targeted and quantificational foraminoplasty device, may help to overcome these anatomical limitations. In this study, we assessed the efficiency and the short-term effects of PTED with ZESSYS at the L5–S1 level.

**Methods:**

Between January and August of 2018, fifty-six patients with lumbar disc herniation at the single level of L5–S1 and who underwent percutaneous transforaminal endoscopic discectomy were enrolled in this retrospective cohort study. They were segregated into the transforaminal endoscopic surgical system (TESSYS) group and the ZESSYS group. The puncture time, foraminoplasty time, decompression time, and fluoroscopy time were evaluated for operation efficiency. Clinical outcomes were assessed by the visual analog scale (VAS) score and Oswestry Disability Index (ODI) score. The MacNab criteria were used to evaluate patient subjective satisfaction at 12-month follow-up postoperatively.

**Results:**

The average puncture time (5.29 ± 2.05 min), foraminoplasty time (12.82 ± 2.52 min), and fluoroscopy time (26.29 ± 5.96 s) were all significantly shorter in the ZESSYS group than in the TESSYS group (average puncture time 8.07 ± 3.13 min, *p* < 0.01; foraminoplasty time, 17.18 ± 2.92 min, *p* < 0.01; fluoroscopy time, 34.73 ± 6.86 s; *p* < 0.01). No significant differences were observed between the 2 groups in the decompression time (*p* = 0.057). The VAS score of low back pain and leg pain, as well as the ODI score, improved at all time points postoperatively compared with preoperative, in both the TESSYS group and the ZESSYS group (*P* < 0.05). There were no significant differences in the VAS score of low back pain, VAS score of leg pain, and ODI score between the TESSYS group and the ZESSYS group at the same time points (*P* > 0.05). According to the MacNab criteria, the excellent and good rate at 12-month follow-up postoperatively was 85.7% in the TESSYS group and 89.3% in the ZESSYS group (*P* > 0.05).

**Conclusion:**

The targeted and quantificational foraminoplasty device named ZESSYS was more efficient in the puncture and foraminoplasty procedures, effectively protecting the exiting nerve and minimizing the level of radiation exposure. The device is efficient and safe for PTED in treating lumbar disc herniation at the L5–S1 level.

## Introduction

Lumbar disc herniation (LDH) is a frequently occurring disease of the spine and leads to economic and medical burdens on families and society [1, 2]. Conservative treatment for LDH is effective, but a considerable number of patients will ultimately receive surgical treatment [3, 4]. Open microdiscectomy has been considered as the standard for surgical treatment for LDH until now. However, in recent decades, percutaneous transforaminal endoscopic discectomy (PTED), via a posterolateral approach, has gained popularity in clinical practice due to advantages such as little trauma, few scar, a rapid recovery, and a short hospital stay [5–9]. For this minimally invasive surgery, a working cannula with a diameter of 7.5 mm needs to be introduced safely to the space in front of the spinal dural sac via the posterolateral direction, and foraminoplasty often needs to be performed to enlarge the intervertebral foramen. The superior articular process (SAP) is the main obstacle for foraminoplasty. Transforaminal endoscopic surgical system (TESSYS) is a classic technique that was invented by Hoogland et al. [10] in 2006. Graded isocentric trephine and accompanying instruments are used to cut the upper and ventral aspects of the SAP. The intervertebral foramen is widened gradually so that the working cannula and rod-shaped endoscope can enter the epidural space. Extruded and sequestered discs can be resected and the region of foraminal stenosis can be decompressed directly. However, in this procedure, the isocentric trephine makes contact with the exiting nerve root, traversing the nerve root and para-foramen soft tissue, which is risk and arising concerns of damage to nerves [11]. In addition, at the L5–S1 level, the unique anatomy involving a high iliac crest, sacral ala, large facet joint, large L5 transverse process, narrowed disc space, and narrowed foramen can complicate the foraminoplasty process. These anatomical obstacles and inter-individual variability in the anatomy make PTED technically challenging [12]. PTED is more difficult to perform at this level in particular [13], even for skilled and experienced surgeons.

ZESSYS is a novel targeted and quantificational foraminoplasty device that originated from a modified version of the traditional TESSYS technique. The novel effective foraminoplasty tool was designed by Yue Zhou et al. from the Second Affiliated Xinqiao Hospital of Army Medical University in Chongqing, China. The hypothesis is that it would be easier to implement and may help overcome the anatomical limitations at the L5–S1 level. In this study, the novel targeted and quantificational foraminoplasty device was used to perform PTED at the L5–S1 level. Meanwhile, its foraminoplasty efficiency and the short-term clinical outcomes were assessed.

## Materials and methods

### Patients

From January to August of 2018, a total of fifty-six patients with lumbar disc herniation who were treated in the Department of Orthopedics in Xinqiao Hospital at the Army Medical University were enrolled in the study. The inclusion criteria were as follows: (1) lumbar disc herniation at the single level of L5–S1 confirmed by preoperative magnetic resonance imaging (MRI) and computed tomography (CT) scans; (2) low back pain accompanied by sciatica and corresponding radiculopathy signs such as motor weakness, sensory deficiency, and the presence of abnormal reflex (more than one of the above signs); (3) a lack of improvement after conservative treatment for at least 12 weeks; and (4) an age of 18–70 years. The exclusion criteria were as follows: (1) central stenosis (less than 10 mm) or lateral recess stenosis (less than 3 mm) confirmed by MRI and CT scans, (2) spinal instability confirmed by dynamic radiographs, (3) calcified disc herniation, (4) far lateral disc herniation, (5) a history of surgery in the same segment, (6) cauda equina syndrome, (7) BMI > 28 kg/m^2^, and (8) the unwillingness or inability to participate in the treatment. All 56 patients were averaged to the TESSYS and the ZESSYS group. All the patients completed the follow-up at 12 months postoperatively. All the study procedures were approved by the Ethics Committee of Xinqiao Hospital, at the Army Medical University. The procedures were performed by a single skilled and experienced surgeon.

### Surgical methods

#### Surgical instruments

The spine transforaminal endoscope system (TESSYS instrument system: Joimax, Inc., Irvine, CA, USA) and tip-flexible electrode bipolar radiofrequency system (Elliquence LLC, Baldwin, New York, USA) were used in the two groups. In the ZESSYS group, the patented ZESSYS instrument instead of the conventional grade guide rod and trephine was used for targeted and quantificational foraminoplasty. ZESSYS is a dual-cannula instrument that has 4 graded sizes. The thinner cannula contains Kirschner wire for fixation and the other larger cannula for foraminoplasty by using a trephine. The detailed characteristics of ZESSYS including the external form, initial application, and advantages were first detailed by Ao et al. [14]. C-arm fluoroscopy was used for radiograph imaging in both groups.

#### Surgical procedures

##### TESSYS

The procedure was performed with local anesthesia combined with analgesic drugs. The patient laid on the radiolucent table in the prone position, and a soft and frame cushion was placed beneath the abdomen to reduce the degree of lumbar lordosis and avoid abdominal compression. The puncture point was 12–14 cm from the middle line and a short distance from the iliac crest if the iliac crest obstacle to the predetermined trajectory. Local infiltration anesthesia was induced by 0.5% lidocaine around the puncture point. An 18-gauge needle was inserted toward the upper and ventral surface of the SAP for stepwise and additional local anesthesia. After the cranial part of the SAP was crossed, the tip of the 18-gauge needle was pushed gently to the ideal epidural position toward the middle line of the vertebral canal under anteroposterior imaging and toward the vertebral posterior superior margin under lateral imaging to the greatest extent possible. If needed, an adjustment toward the herniated disc was performed. At that point, the 18-gauge needle was withdrawn and replaced by a guidewire. Over the guidewire, a stab incision measuring approximately 1 cm was made, and stepwise guiding and dilatation rods were introduced. Subsequently, foraminoplasty was performed after the ventral side of SAP was partially removed with the expansion tube, guide rod, and trephine in sequence. After surgical access was established, a working channel slope was placed close to the intervertebral disc. In all cases, the herniated disc and the extruding or sequestrated disc fragment could be observed. Then, discectomy and nerve root decompression were performed in the same manner as was the routine PTED procedure. After decompression, the surgical instruments were pulled out, and the wound was sutured.

##### ZESSYS

This procedure was also performed with local anesthesia combined with analgesic drugs. The protocol used before the puncture step was identical to that used in the TESSYS group. An 18-gauge needle was inserted into the foramen and across the safe triangle stepwise. During this step, the needle was inserted along the ventral aspect of the SAP to prevent exiting nerve root injury. The needle was then replaced by a 1-mm guidewire with a stab incision measuring approximately 1 cm. After traditional graded dilation, the 1-mm guidewire and the smallest guide rod were replaced by a 2.5-mm Kirschner wire which was slightly hammered across the safe triangle and fixed on the superior-lateral part of the posterior aspect of the distal vertebra. Based on the location of the Kirschner wire tip on the anteroposterior and lateral view, the extent of deviation to the target point was assessed and the double-cannula device with a properly predetermined size was selected. As a rule, selection of larger size can provide more ventral bony ablation of the SAP and will obtain final intracanal positioning that is closer to the middle line on the anteroposterior view. Following the Kirschner wire, the suitable double-cannula device was inserted until the larger cannula reached the SAP through the incision. Afterwards, the double-cannula device was rotated and centered on the Kirschner wire to determine the ideal starting point and trajectory to the target point on the anteroposterior and lateral view. Foraminoplasty was then performed by a trephine, and part of the ventral side of the SAP was cut and removed. If needed, the foramen was widened further by a larger graded cannula or another rotational motion. Then, discectomy and nerve root decompression were performed in the same manner as in the routine PTED procedure. After decompression, the surgical instruments were pulled out, and the wound was sutured.

### Statistical analysis

Statistical analyses were performed using the SPSS 17.0 statistical package (SPSS, Inc., Chicago, IL, USA). The two independent samples t-test, paired t-test, chi-square test, and Mann-Whitney U test were used for data analyses. The measurement data are presented as the means ± standard deviations. For all analyses, a p value less than 0.05 was considered statistically significant.

## Results

### Patient demographic data

Fifty-six patients (TESSYS, 28 patients; ZESSYS, 28 patients) who underwent PTED surgery from January to August of 2018 were enrolled in this study. The baseline demographics for both groups are shown in Table 1. The grade of foraminal stenosis on the operative side at L5–S1 was classified according to the report of Lee et al. [15]. The grade of iliac height at L5–S1 was classified according to the report of Choi et al. [16]. No significant differences were observed between the 2 groups (Table 1).

**Table 1:** Patient demographic data for the TESSYS and ZESSYS groups

	TESSYS	ZESSYS	*p* value
Age, years	52.54 ± 9.10	49.21 ± 10.38	0.208
Sex, male:female	18:10	15:13	0.415
ASA criteria, I:II	20:8	18:10	0.571
BMI (kg/m^2^)	22.28 ± 2.69	21.99 ± 2.22	0.659
Smoker, yes:no	16:12	17:11	0.786
Duration of symptoms, weeks	15.21 ± 2.54	16.04 ± 2.96	0.271
Herniation type, contained:uncontained	9:19	11:17	0.577
Foraminal stenosis grade (L5–S1) 0:1:2:3	19:9:0:0	17:10:1:0	0.523
Iliac height grade 1:2:3:4:5:6	0:0:3:8:10:7	0:0:1:9:11:7	0.730

*ASA* American Society of Anesthesiologists, *BMI* body mass index

### Clinical outcomes

As shown in Table 2, the average puncture time (5.29 ± 2.05 min), foraminoplasty time (12.82 ± 2.52 min), fluoroscopy time (26.29 ± 5.96 s), and total surgery time (63.14 ± 7.76 min) in the ZESSYS group were significantly shorter than those in the TESSYS group (average puncture time 8.07 ± 3.13 min, *p* < 0.01; foraminoplasty time, 17.18 ± 2.92 min, *p* < 0.01; fluoroscopy time, 34.73 ± 6.86 s; *p* < 0.01; total surgery time 74.21 ± 12.16 min, *p* < 0.01). No significant differences were observed between the 2 groups in the decompression time (*p* = 0.057).

**Table 2:** Comparison of the intraoperative outcomes between the TESSYS and ZESSYS groups

	TESSYS	ZESSYS	*p* value
Puncture time (min)	8.07 ± 3.13	5.29 ± 2.05	< 0.01
Foraminoplasty time (min)	17.18 ± 2.92	12.82 ± 2.52	< 0.01
Fluoroscopy time (s)	34.73 ± 6.86	26.29 ± 5.96	< 0.01
Decompression time (min)	48.96 ± 9.02	45.04 ± 5.65	0.057
Total surgery time (min)	74.21 ± 12.16	63.14 ± 7.76	< 0.01

As shown in Table 3, the VAS scores for low back pain and leg pain, as well as the ODI score, improved at all time points postoperatively, both in the TESSYS group and the ZESSYS group (*p* < 0.05). There were no significant differences in the VAS score for low back pain, VAS score for leg pain, ODI score, or MacNab criteria between the TESSYS group and the ZESSYS group at the same time points (*p* > 0.05). The excellent and good rate according to the MacNab criteria at 12 months postoperatively was 85.7% in the TESSYS group and 89.3% in the ZESSYS group.

**Table 3:** Comparison of the clinical outcomes of the TESSYS and ZESSYS groups

	TESSYS	ZESSYS	*p* value
VAS score for low back pain			
Preoperative	3.60 ± 0.95	3.38 ± 0.69	0.310
Postoperative	2.20 ± 0.92*	2.36 ± 1.03*	0.541
12 months	1.48 ± 1.02*	1.30 ± 0.84*	0.467
VAS score for leg pain			
Preoperative	5.91 ± 1.23	6.17 ± 1.61	0.505
Postoperative	2.10 ± 0.97*	2.25 ± 0.86*	0.532
12 months	1.22 ± 0.70*	1.10 ± 0.84*	0.572
ODI score			
Preoperative	59.36 ± 8.33	61.57 ± 7.89	0.312
Postoperative	15.50 ± 4.48*	16.07 ± 4.82*	0.648
12 months	12.50 ± 3.06*	12.07 ± 3.67*	0.637
MacNab criteria (12 months)			
Excellent	17	19	0.569
Good	7	6	
Fair	4	3	
Poor	0	0	

*VAS* visual analog scale, *ODI* Oswestry Disability Index

*Compared with preoperatively, *P* < 0.05

### Operation complications

All the patients in the two groups were successfully decompressed with immediate nerve relief. No cases required conversion to an open procedure, and no major complications such as dural tears, infections, or vascular injuries were observed. One patient in the TESSYS group developed postoperative dysesthesia (POD), which was considered to be caused by irritation to the dorsal root ganglion. With temporary conservative treatment, the symptoms disappeared. No cases of recurrent herniation were observed at the 12-month follow-up.

## Discussion

PTED technic has been an effective and safe procedure for LDH [17–20]. Hoogland et al. firstly invented the TESSYS technique, in which a graded trephine was used to enlarge the foramen gradually and the working channel slope was placed close to the intervertebral disc. However, the extruded and sequestered disc fragments are accessible only when the operating instruments are placed in the optimal trajectory. Accuracy was highly demanded, especially for the puncture and foraminoplasty procedure. At the L5–S1 level, the high iliac crest is a major block for the PTED procedure. The high iliac crest in some cases limited the ideal angle of puncture and channel implantation. Failure of puncture was reported in many cases. The other anatomy includes sacral ala, large L5 transverse process, large facet joint, narrowed disc space, and narrow foramen which also complicate the puncture [21], foraminoplasty, and channel indwelling processes of PTED. The unique anatomy at the L5–S1 level leads to a steeper learning curve [22] and challenges, even for skilled surgeons. In addition, the gradual trephine is isocentric, which increases the risk of damage to nerve roots. Most POD cases due to dorsal root ganglia stimulation or injury have been reported to occur in L5–S1 during the PTED procedure [23, 24]. Therefore, the interlaminar approach and transiliac approach endoscopic surgery were used to treat LDH at the L5–S1 level. But these two techniques had defects. It was hard to resect lateral and far lateral herniated nucleus pulposus through the interlaminar approach. The transiliac approach would cause additional damage to the iliac and had a potential risk of the sacroiliac joint and peripheral soft tissue injury. In addition, the accurate puncture point of the iliac and safety working channel were difficult to position. To address the above problem, the ZESSYS, a targeted and quantificational foraminoplasty device with a double cannula, is conducive to overcoming the anatomic limitations at the L5–S1 level via the classical posterolateral approach.

**Fig. 1:**
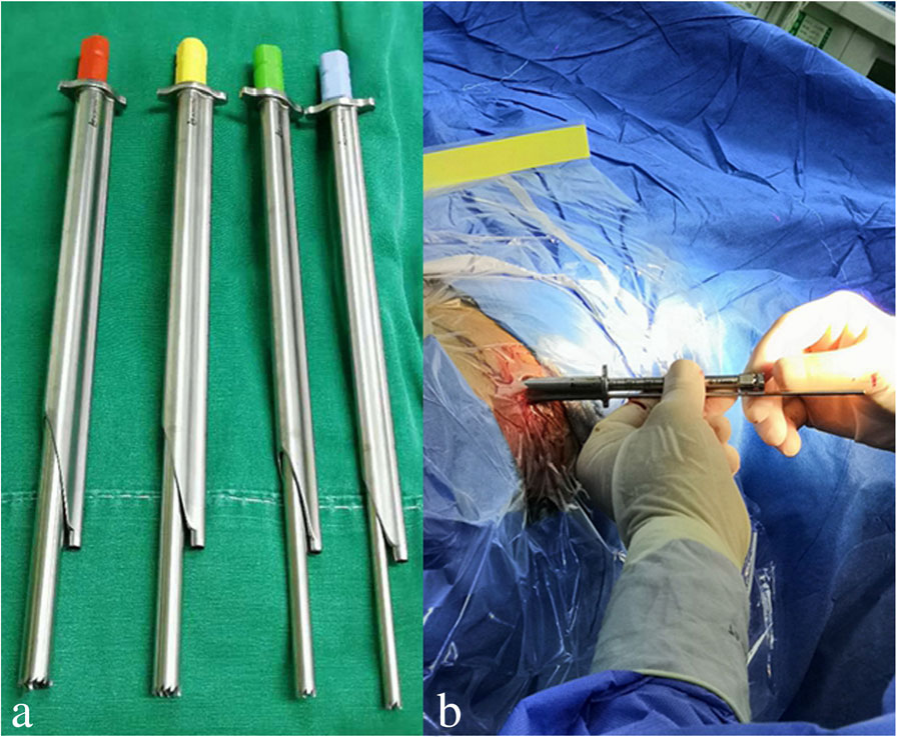
Composition of the ZESSYS device: **a** 4 graded dual-cannula size. **b** The thinner cannula containing the Kirschner wire for fixation and the other larger cannula that was used with a trephine for foraminoplasty

**Fig. 2:**
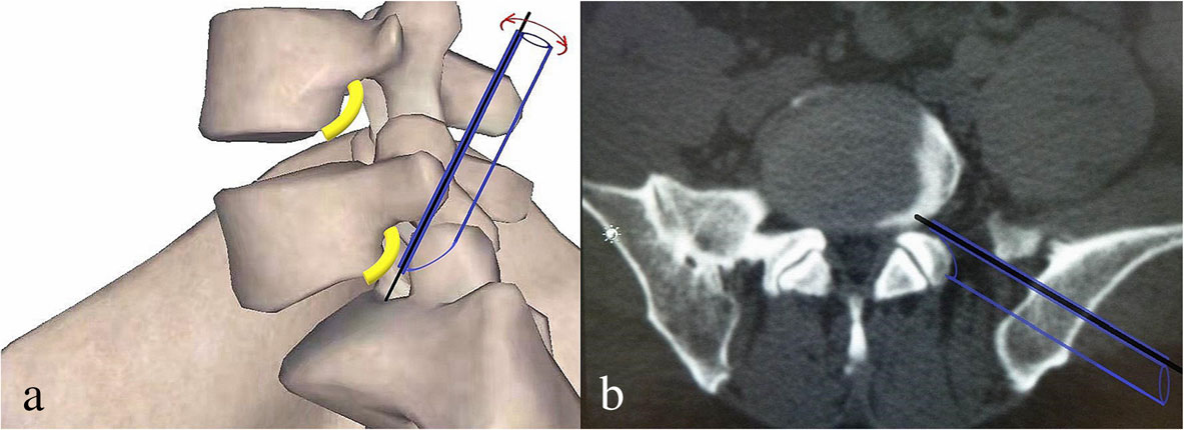
Schematic diagram of the ZESSYS device: The black line segment represents the Kirschner wire, acting as a steady pivot for the double-cannula device. The purple graphics represents the double-cannula device that can be rotated with the Kirschner wire as a center to find proper foraminoplasty trajectory and achieve quantificationally decompression by selection of four different sizes

Firstly, it can reduce the difficulties of acupuncture. In the conventional TESSYS technique, an 18-gauge needle is inserted toward the middle line of the vertebral canal under AP imaging and toward the vertebral posterior superior margin under lateral imaging, and subsequently, foraminoplasty is performed to achieve a sufficient level of surgical access for PTED. If needed, the targeted point can be adjusted for special extruded and sequestered disc fragments. When the high iliac crest, large L5 transverse process, and large facet joint obstruct the trajectory at the L5–S1 level, the ideal or targeted puncture point is time-consuming, difficult, or even inaccessible to reach, and the foraminoplasty time and fluoroscopy time increase correspondingly. ZESSYS is a dual-cannula adjustment instrument with a thin cannula containing a Kirschner wire for orientation and a larger cannula for bony abrasion by a trephine (Fig. 1). Primary puncture with an 18-gauge needle was only demanded to cross the safe triangle area stepwise; after the guidewire was introduced and graded dilation was performed, the traditional guidewire was replaced with a 2.5-mm Kirschner wire and slightly hammered to fix on the posterior aspect of the distal vertebra, which could guide the following introduction of the dual-cannula device for foraminoplasty. The trajectory of the Kirschner wire did not need to strive for the accurate requirement of the TESSYS technique; it was necessary to insert the wire between the exiting nerve root and the SAP, and it could be fixed at any position near the superior-lateral part of the posterior aspect of the distal vertebra. The double-cannula system takes advantage of rotation and can be easily adjusted to find a proper and targeted entry point on the SAP, which can compensate for the Kirschner wire primary puncture point (Fig. 2). In addition, depending on the last trajectory of the Kirschner wire, size 4 of the graded dual-cannula size may be selected for selective resection of the SAP for quantificational foraminoplasty (Figs. 1 and 2). However, as a rule, an extruding or sequestrated disc fragment is within the scope of the working channel’s coverage. In addition, if predetermined effective access to enter or across the safe triangle area is hard to achieve or inaccessible by using an 18-gauge needle in the case of a large L5 transverse process, large facet joint, narrowed disc space, or narrow foramen impeding the predefined trajectory, after replacement, the Kirschner wire can take place of the needle for the puncture process as it is more rigid and easier to adjust. In particular, the use of a Kirschner wire with a 2.5-mm diameter for trajectory adjustment and fixation in the safe triangle area increases the risk of nerve root damage, so when the Kirschner wire advances into the foramen, it should cling to the ventral aspect of the SAP, and the patient’s responses should be taken into consideration to avoid possible nerve root and adjacent soft tissue injury. If possible, the tip of the Kirschner wire should be fixed between the inner side of the SAP and the middle line in the anteroposterior view, thereby allowing a small amount of abrasion with the upper articular process.

**Fig. 3:**
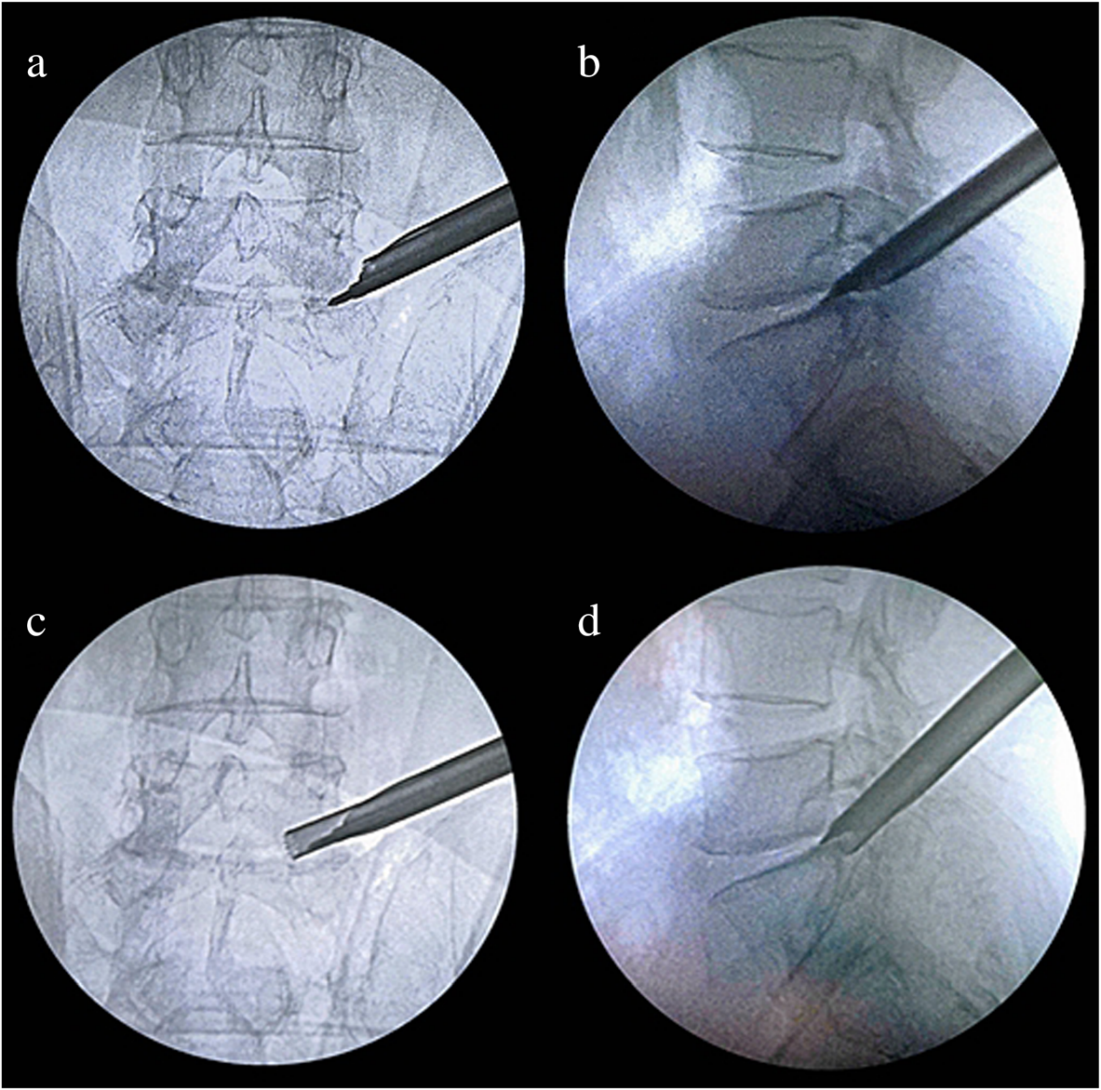
The targeted and quantificational foraminoplasty process: **a**, **b** Following Kirschner wire insertion, the double-cannula device with a properly predetermined diameter was inserted and rotated for a predetermined trajectory. **c**, **d** Foraminoplasty was performed by a trephine; the exiting nerve root was excluded from the working zone and the Kirschner wire acted as a steady pivot

**Fig. 4:**
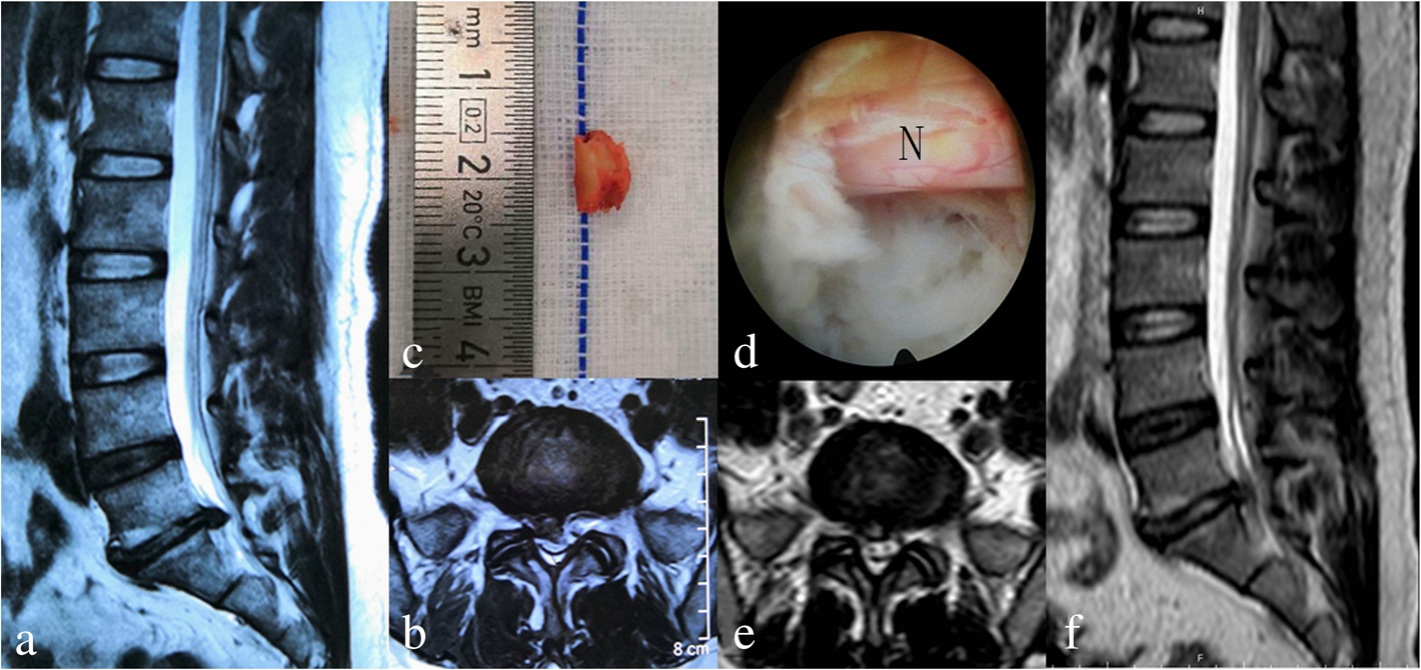
One case in the ZESSYS group. **a**, **b** A 57-year-old female patient diagnosed with lumbar disc herniation at L5–S1 and underwent PTED with the ZESSYS device. **c** Part of the ventral side of the SAP was cut and removed during the foraminoplasty procedure. **d** The S1 nerve root (N) was clearly visualized. **e**, **f** Postoperative (3 days after surgery) MRI scan showing good decompression and incomplete annulus restoration

Secondly, the ZESSYS device can improve the efficiency and safety of foraminoplasty. As mentioned above, among the anatomical limitations at the L5–S1 level, the iliac crest is considered a major obstacle [25, 26]. When the iliac crest is located above the middle of the L5 pedicle in the lateral radiograph, foraminoplasty may be required [27]. In addition, if the working spaces are limited by the other anatomical limitations, foraminoplasty is also necessary. In the conventional TESSYS technique, the foramen is widened gradually by an isocentric trephine. During the process of foraminoplasty, the trephine blade makes close contact with foramen soft tissue and nerve roots, leading to a risk of damage, especially for the L5–S1 level with a narrowed disc space and intervertebral foramen than the other levels. During the PTED process with ZESSYS, a 2.5-mm Kirschner wire is passed between the SAP and exiting nerve root and fixed on the posterior aspect of the distal vertebra, which acts as a steady pivot for the double-cannula device. When the predefined cannula is inserted to dock on the SAP at posterior orientation, it can be easily rotated to find the proper trajectory and achieve quantificationally decompression. In addition, the beveled design cannula is placed next to the ventral side of the SAP, excluding the exiting nerve root from the working zone of the trephine and protecting the exiting nerve root from damage. If needed, the foramen can be enlarged by a second cannula rotation. The double cannulas greatly reduce the difficulty of foraminoplasty and enable foraminoplasty to be performed more precisely (Figs. 3 and 4).

According to our study, the ZESSYS device is more efficient than the TESSYS device in the puncture and foraminoplasty procedures. The fluoroscopy time decreased with the application of ZESSYS, which was beneficial for both the patients and surgeons. The preliminary postoperative outcomes seemed to be equal between the ZESSYS group and TESSYS group. However, 1 patient in the TESSYS group experienced slight dysesthesia after surgery which was a common complication of PTED [18, 23, 28]. There were no cases of severe complications in the ZESSYS group. One of the reasons for this result might be that the new instrument not only effectively widened the foramen but also effectively protected the nerve root.

Nevertheless, our study has some limitations. Firstly, the sample sizes of the two groups were small, and the follow-up time was short. The clinical outcome was preliminary, and a larger study needs to be conducted in the future to verify the reliability of the ZESSYS system. In addition, at the L5–S1 level, the unique anatomical limitations were triaxial and complicated, and there were no specific protocols for the evaluation of the anatomical limitations. Therefore, in this study, we analyzed two major anatomical limitations between the two groups, which may not be enough and may affect the statistical correctness.

## Conclusion

The double-cannula device named ZESSYS is effective and safe for PTED for L5–S1 disc herniation. It was more efficient in the puncture and foraminoplasty procedure, as well as protected the exiting nerve effectively and minimized the degree of radiation exposure.

## Data Availability

All data generated or analyzed during this study are included in the article.

## Abbreviations

PTED: Percutaneous transforaminal endoscopic discectomy
LDH: Lumbar disc herniation
VAS: Visual analog scale
ODI: Oswestry Disability Index
SAP: Superior articular process
POD: Postoperative dysesthesia
MRI: Magnetic resonance imaging
CT: Computed tomography
TESSYS: Transforaminal endoscopic surgical system
